# Formation and Maintenance of the Natural Bypass Vessels of the Brain

**DOI:** 10.3389/fcvm.2022.778773

**Published:** 2022-03-22

**Authors:** Tijana Perovic, Christoph Harms, Holger Gerhardt

**Affiliations:** ^1^Integrative Vascular Biology, Max-Delbrück-Center for Molecular Medicine in the Helmholtz Association, Berlin, Germany; ^2^DZHK (German Center for Cardiovascular Research), Partner Site Berlin, Berlin, Germany; ^3^Center for Stroke Research Berlin with Department of Experimental Neurology, Charité Universitaetsmedizin Berlin, Berlin, Germany; ^4^Einstein Center for Neurosciences Berlin, Charité-Universitaetsmedizin Berlin, Berlin, Germany

**Keywords:** collateral, cerebrovasculature, development, maintenance, ischemic disease

## Abstract

Ischemic diseases are the leading cause of death and disability worldwide. The main compensatory mechanism by which our body responds to reduced or blocked blood flow caused by ischemia is mediated by collateral vessels. Collaterals are present in many healthy tissues (including brain and heart) and serve as natural bypass vessels, by bridging adjacent arterial trees. This review focuses on: the definition and significance of pial collateral vessels, the described mechanism of pial collateral formation, an overview of molecular players and pathways involved in pial collateral biology and emerging approaches to prevent or mitigate risk factor-associated loss of pial collaterals. Despite their high clinical relevance and recent scientific efforts toward understanding collaterals, much of the fundamental biology of collaterals remains obscure.

## Introduction

Collateral vessels are anatomically defined as inter-tree anastomoses cross-connecting adjacent arterial trees (1, 2).

Functionally, they represent a specialized network of endogenous bypass vessels, which serve to partially attenuate hypoperfusion or ischemic injury following blockage of an artery. Collateral retrograde perfusion from adjacent territories may provide transient or permanent endogenous protection against ischemic injury in various organs (caused by ischemic stroke, coronary atherosclerosis, myocardial infarction, peripheral artery disease, etc.). However, the extent to which collaterals endow individuals with protection against occlusive disease varies greatly and directly impacts clinical outcome (3, 4). Naturally occurring differences in the number and diameter of collateral vessels as well as their ability to rapidly increase their diameter upon arterial vessel occlusion limit the protective capacity of collaterals (5). In humans, angiography of patients suffering from acute middle cerebral artery (MCA) occlusion show that retrograde perfusion of the ischemic MCA territory downstream from the occlusion *via* pial collaterals exhibits significant variation among individuals. Good collateral flow correlates with improved likelihood of major reperfusion, reduced infarct expansion and other favorable outcomes: infarct volume and modified Rankin scale scores at discharge are significantly lower for patients with better pial collaterals (angiographically assessed), while the National Institutes of Health Stroke Scale (NIHSS) score and collateral flow scores show an inverse relationship. Nowadays, MRI diffusion and perfusion imaging together with angiographic collateral scoring during acute cerebral ischemia show that patients with good collaterals have larger areas with only mild hypoperfusion and reduced infarct growth within the penumbra (6).

In an effort to standardize the terminology around collateral vessels, Faber and colleagues (1) define collaterals as *naturally occurring artery-to-artery or arteriole-to-arteriole anastomoses present in healthy tissues that increase their anatomic diameter, i.e., outwardly remodel, in obstructive disease*. Furthermore, they describe two distinct types of collateral vessels:

■ Collateral arteries, which are, in fact, artery-to-artery anastomoses and occur in anatomically similar locations among humans and other mammals. Due to their common anatomical location, they often have a defined name (e.g., superior ulnar collateral artery, anterior and posterior communicating arteries/collaterals of the circle of Willis). Mature healthy collateral arteries exhibit minimal or no tortuosity, have a considerably smaller capacity to increase their lumen in obstructive disease and form differently from microvascular collaterals.
■ Microvascular collaterals are arteriole-to-arteriole anastomoses that cross-connect a small fraction of distal-end arterioles in the crowns of adjacent arterial trees. These vessels in healthy humans and animals average <100 microns in diameter. Interestingly, they are completely absent in the mouse retinal circulation. Examples are: pial (leptomeningeal) collaterals of the brain and spinal cord, coronary collaterals, collaterals of the skeletal muscle and skin. They are characterized by significant tortuosity in healthy young adults and their inherent capacity to enlarge their lumen 5–10-fold upon occlusive disease. Between different inbred mouse strains, there exists a large genetic background variability in collateral number, diameter and remodeling capacity. Considering that collateral arteries have distinct names, usually the term *collateral* implies the microvascular collaterals of a given tissue/organ.

## Mechanisms of Formation—Brain vs. Heart

In mice, pial collaterals have been reported to begin forming between embryonic day 13.5 (E13.5) and 15.5 (E15.5), with the peak collateral formation at E18.5 (7, 8). The pial vasculature matures between E18.5 and approximately postnatal day 21 (P21), involving the pruning of a variable proportion of nascent collaterals. The remaining collaterals undergo wall maturation, increase their diameter and length and acquire their characteristic tortuosity. The process of collateral formation during embryonic and postnatal development to yield the collateral extent present in the healthy adult tissue is termed collaterogenesis (1). To date, many details on the mechanism of collateral formation remain unclear: collaterals present in the adult may arise either by 1) retention or transformation of a capillary vessel(s) present early in embryonic/early postnatal development (pre-existing arteriolar connections) or by 2) sprouting from established arterioles to form novel inter-arteriolar connections. One current hypothesis suggests that pial collaterals form *via* arteriolar sprouting during late gestation (8). This is based on the exclusion of intussusception as a forming mechanism, as no intussusceptive pillars could be observed *via* confocal or scanning electron microscopy. Additionally, the authors identified a vessel which appears to be sprouting from a pial arteriole.

One angiographic study (9) shows that in human embryonic hearts (between 19 and 39 weeks, from the mid-second trimester until the end of the third trimester), collateral coronary arteries are already present, ranging between 3 and 50 micrometers in diameter. It has, in fact, been confirmed that human hearts have inherent collateral vessels in individuals with no previous occlusion (individuals with *normal coronary arteries*) (10). A more recent report investigated the presence of pre-existing collaterals in the mouse heart using various techniques, namely: angiographic casting, casting with low-viscosity Microfil or with high pressure, casting after minimizing resistance, perfusion with Evans-blue PBS, staining with Isolectin and ephrin-B2^Lacz/+^ on two different backgrounds (B6 and BALB/c) (11). This study alongside others indicates that in the mouse heart, collateral coronary arteries form only upon vascular occlusion (also termed neo-collateral formation, *de novo* collateral formation in adults), and once again, determine the clinical outcome of infarction. Patients with significant collateral coronary arteries can survive having one or two completely occluded native coronary arteries and exhibit normal heart function. Most studies of embryonic microvascular collaterogenesis in the past two decades have focused on microvascular collaterals of the brain and hindlimb. A genetic lineage tracing study by He et al. (12) identified that upon myocardial infarction in adult mice, new coronary collateral vessels are formed from existing arteries. Briefly, the genetic lineage tracing method uses a cell-type specific Cre driver mouse line, which in this case is the capillary-specific Apln-CreER. Cre is expressed as a fusion protein to the mutated estrogen receptor (ER) to mediate activation in a conditional fashion by treatment with Tamoxifen. Such a mouse line is then crossed with a Cre-dependent reporter mouse line, which can be pharmacologically activated by Tamoxifen as it harbors a stop cassette flanked by loxP sites that are Cre-responsive (13). This allows for the reporting of certain cell types and their permanent lineage tracing over time, as expression of the reporter is irreversible once activated, and passed on to daughter cells when they divide. By genetic tracing of capillary-specific Apln-CreER cells, the authors showed that a mid-embryogenesis Tamoxifen induction with Apln-CreER will label both coronary arteries and capillaries at P7. However, if Apln-CreER was induced only after birth, at P1 or in the adult mouse, only the coronary microvasculature is labeled. This implies that the embryonic coronary capillaries significantly contribute to the formation of coronary arteries. When myocardial infarction was induced in adult-induced Apln-labeled hearts, no contribution of capillaries was found to the newly formed collaterals. Kristy Red-horse and colleagues (14) looked specifically into the mechanisms of formation of collateral coronary arteries and found that upon permanent ligation of the left coronary artery (LCA) at P2 neonates, arterial endothelial cells migrate from existing arteries, along capillaries and reassemble into collateral arteries, which the authors termed *artery reassembly*. Moreover, this process was largely dependent on the chemokine CXCL12—CXCR4 receptor signaling axis. In adult mice, the *artery reassembly* after myocardial infarction could be triggered by administering a single dose of CXCL12.

## Pial Collaterals—Pathways That Play a Role in Their Development and Maintenance

Although the question of whether collaterals possess a truly unique transcriptional and proteomic profile remains open, several molecular factors have been shown to affect collateral formation, maturation, maintenance and response to ischemia.

### Formation

Embryonic collateral formation is dependent on VEGF signaling. In two mouse strains which exhibit large differences in collateral density, namely C57BL/6 and BALB/c, *Vegfa* expression was higher in the C57Bl/6 (the strain with higher collateral density) than in BALB-c mice (7). Functionally, hypomorphic *Vegf*^*lo*/+^ embryos developed almost no collaterals. Inducible, global knockdown of either *Vegfa* or *Flk1* (VEGFR2 gene) impairs embryonic collateral formation. However, endothelial specific inducible *Vegfa* deletion had no effect on collateral formation, suggesting that paracrine VEGF signaling is relevant in collateral formation (8). Notch signaling works in conjunction with VEGF signaling in the process of endothelial tip cell selection and sprout formation. Membrane-bound Notch becomes active only upon two cleavage steps (ADAM sheddases participate in the 1st step, gamma secretase in the 2nd step), which allow for its translocation to the nucleus and target gene activation (15). Both endothelial-specific *Adam10* knockdown and pharmacological inhibition of gamma-secretase lead to an increase in embryonic collateral formation (8). The authors suggested that paracrine VEGF through the endothelial VEGFR2-ADAM10-Notch signaling pathway is crucial for embryonic development of pial collaterals, and when altered, permanently changes collateral density in the adult.

Intercellular communication to Notch is transmitted *via* Delta-like 4 (Dll4). Dll4-Notch signaling is a pathway implicated in the regulation of arterial identity and angiogenic sprouting (15–17). Dll4 is a transmembrane ligand of Notch receptors, selectively expressed in arterial and angiogenic tip cells during development. Similarly, Dll4-Notch signaling restricts pial collateral artery formation by modulating arterial branching morphogenesis during embryogenesis (18). DLL4 heterozygous mice show an increased number of pial collaterals compared to littermates, whereas the infarct volume upon MCA occlusion remains unchanged. Furthermore, functional recovery and ischemic outcome in stroke and hindlimb ischemia models were not improved in Dll4^+/−^ mice, despite the clear increase in collateral vessel number. The authors speculate that this discrepancy is due to the adverse effects Dll4-Notch loss has on vessel formation and remodeling during development. Together, these results indicate that the protection pial collateral networks provide in ischemic stroke is not only determined by collateral numbers, but also by collateral functionality.

Mouse strains with different genetic backgrounds exhibit wide variation in collateral density, ~80% of which is assigned to a polymorphic region on chromosome 7, *Dce1*. A single gene, *Rabep2*, was identified as responsible for most of the differences in native collateral density. Collateral formation is impaired in *Rabep2*^−/−^ embryos (5). Rabep2 is ubiquitously expressed and associated with vesicular trafficking, particularly in the internalization of cell surface receptors into vesicles which fuse into early endosomes in a Rab4- and Rab5-dependent matter. The embryonic pial plexus of *Rabep2*^−/−^ mice exhibits increased vessel diameter and reduced branching. Moreover, early endosomes are enlarged in E14.5 *Rabep2*^−/−^ mice. *In vitro*, Rabep2-deficiency leads to increased Rab7 co-localization of VEGFR2, indicating that in absence of Rabep2, a higher proportion of internalized VEGFR2 is targeted for degradation (19).

### Maturation

Chloride intracellular channel-4 (CLIC4) is a member of a 7-membrane-spanning family of proteins (CLICs). Knockdown of CLIC4 impairs EC proliferation, as well as formation of EC cords and tubular plexus. Clic4(–/–) mice have reduced native collateral density, which results in more severe infarctions (20). In a follow-up study, the authors have shown that Clic deficiency has no effect on embryonic collaterogenesis, yet leads to reduced mural cell recruitment and excessive pruning of pial collaterals. VEGF-A overexpression in CLIC4-deficient mice partially rescues deficits in perinatal collateral mural cell investment, and fully rescues aberrant perinatal collateral pruning and enlarged infarct volume after stroke in adults (21). Whereas Vegfr2 signaling is involved in both formation and maturation of pial collaterals, other pathways are more confined: Notch signaling seems crucial in collateral formation and CLIC4 in collateral maturation.

Ephrin (Eph) receptors are known to control cell migration, proliferation and mediate responses to guidance/repulsive cues. They have well-identified roles in neuronal development (axon guidance, neural crest migration, etc.) (22). EphrinB2 and EphB4 null mice show defects in arterio-venous patterning. Ephrin-B2, an Eph family transmembrane ligand, marks arterial but not venous endothelial cells from the onset of angiogenesis whereas Eph-B4, a receptor for ephrin-B2, marks veins but not arteries. Interestingly, endothelial-specific EphA4 deletion leads to an early postnatal increase in collateral number, but not diameter (23). By P21, this number lowers to wild-type values. Further work suggests that EphA4 acts as a major suppressor of pial collateral remodeling, as well as cerebral blood flow (CBF) and functional recovery after permanent middle cerebral artery occlusion, by acting as a negative regulator of Tie2 receptor signaling (24).

### Signaling in the Collaterals of the Heart

Molecular effectors and pathways responsible for collateral formation in the mouse heart have only started to be studied. In 2015, Zhang and Faber showed the dependency of neo-collateral formation on MCP1—CCR2 signaling. MCP1 is released from cardiomyocytes, endothelial, smooth muscle cells and a variety of hematopoietic cells types and binds CCR2 receptors which are present on monocytes, CD4 T-cells, endothelial cells and others. Mice lacking either MCP1 or CCR2 exhibited reduced neo-collateral formation and increased infarct volume (11). Interestingly, a recent cohort study showed that low matrix metalloproteinase-9 (MMP-9) and high MCP1 levels are associated with good pretreatment collateral status in patients suffering from acute ischemic stroke with large vessel occlusion (25).

The chemokine CXCL12, also known as SDF1, has chemotactic and mitogenic activity on many cell types (26). CXCL12 signaling has an important role in vasculogenesis, including endothelial cell migration, arterial-nerve alignment and mediation of plexus connections to systemic arteries. CXCL12 primarily acts through the G protein coupled receptor CXCR4; global mouse knockouts of Cxcl12 or of Cxcr4 die shortly before birth with vascular deficiencies in the gut, kidney, and skin, and with a number of additional hematopoietic and neural defects (27). Cxcl12 is important for guiding coronary EC migration during embryonic development. One study identified the CXCR4—CXCL12 axis as necessary for early postnatal collateral formation in response to myocardial infarction. Moreover, coronary collateral development was inhibited upon endothelial Cxcl12 or arterial Cxcr4 deletion. One dose of CXCL12 at the time of adult myocardial infarction stimulated collateral growth. The authors suggest that in this mechanism of *arterial reassembly*, arterial endothelial cells are attracted by a capillary CXCL12 gradient, in order to migrate, expand and establish a novel collateral artery network (14).

## Pial Collaterals—Emerging Concepts in Ischemia: Preventive Conditioning of Collaterals (Role of Exercise, Hypoxia, eNOS Signaling)

The field of pial collateral biology has gained a lot of momentum in the past two decades, yet there are still many unknowns. Important questions are yet to be answered: 1) What prevention measures can be taken to halt or revert the progressive loss (rarefaction) of collaterals in aging individuals? 2) What acute intervention steps can be taken to stimulate the inherent *bypassing* capacity of collaterals upon stroke? 3) What acute intervention steps might stimulate neo-collateral growth in the adult?

A report from Rzechorzek et al. studied the effect of voluntary wheel running, a proxy for aerobic exercise in mice, on the outcome of permanent MCA occlusion in aging mice (26-month-old mice). In this study, the authors compared 3-month-old sedentary mice to 26-month-old sedentary and running mice. Their results indicate that regular aerobic exercise prevents age-induced rarefaction of pial collaterals and associated increase in infarct volume (28). Another interesting report from Zhang et al., examined the impact of hypoxia on adult mice neo-collateral formation. After gradually acclimating mice to lower concentrations of inspired oxygen and maintaining them for 2–8 weeks at 12, 10, 8.5, or 7% inspired oxygen concentrations, the authors observed a correlation between neo-collateral formation and hypoxemia, as well as remodeling of native collaterals and decreased infarct volume after permanent MCA occlusion and hypoxemia. Hypoxia led to an increased expression of *Hif2*α*, Vegfa, Rabep2, Angpt2, Tie2*, and *Cxcr4*. Moreover, neo-collateral formation was abolished in mice lacking Rabep2, and inhibited by conditional knockout of *Vegfa, Flk1*, and *Cxcr4* (29). These results suggest mechanistic links between embryonic collateral formation and neo-collateral formation in adult mice. Whether an increased need for oxygen is enough of a stimulus for adult physiological neo-collateral formation in humans as well is not known.

Additionally, a recent publication indicated that pial collateral cells are endowed with primary cilia more frequently than their neighboring vessels, distal-most arterioles. Moreover, collateral vessels showed an increased expression of *Pycard, Ki67, Pdgfb, Angpt2, Dll4*, and *Ephrinb2* when compared to their neighboring distal-end arterioles. Collaterals were enriched in both eNOS and phospho-eNOS compared to distal-most arterioles (30).

Interestingly, global eNOS KO mice have fewer pial collaterals and worse perfusion capacity upon femoral artery ligation (31). One recent report (32) proposed the cell cycle gene networks as the pathways responsible for the role of eNOS in collateral health and disease. It remains to be seen which of the effects of eNOS loss are specific for the endothelium and what is the role of paracrine signaling in pial collateral response to injury. In rodents, aging correlates with collateral rarefaction (33). According to Wang et al. (31), in a hind-limb ischemia model, aging decreases collateral responsiveness to angiogenic stimuli and increases endothelial and smooth muscle cell susceptibility to apoptosis *via* lack of functional eNOS signaling.

## Concluding Remarks and Outlook

Collateral vessels are a rare gem in vascular biology. They undergo massive remodeling in a matter of days upon an ischemic event, all while maintaining vessel integrity and function. In the brain, an organ of high complexity and metabolic demand and low regenerative capacity, this ability of pial collateral vessels to quickly expand directly determines the volume of the damaged neuronal tissue. Therefore, it is of utmost importance for vascular biologists to understand the fundamentals of collateral formation, maintenance and remodeling in order to harness this knowledge and translate it into generation of more targeted therapeutics. If we understood exactly how pial collaterals form on the levels of brain morphogenesis, individual cell behavior and molecular drivers, we would know more about how to reactivate collateral formation or opening in patients suffering from ischemia with particularly poor prognosis due to collateral rarefaction or low collateral blood flow. In this review, we aimed to highlight the most important findings in collateral biology, in terms of endothelial cellular behavior in developing collaterals as well as in terms of molecular effectors driving collateral formation and maturation. Despite their anatomical and biomechanical uniqueness, we still do not know whether native collaterals are somehow molecularly equipped to adapt to new blood flow requirements so rapidly. Only in recent years have scientists started to understand ways of preserving or increasing the abundance of collaterals in tissues by means of exercise and hypoxic treatment.

Pre-clinical models and animal research is currently highlighting commonalities and differences in heart and brain collaterals, and point toward signaling mechanisms of general importance in vascular formation and remodeling, such as hypoxia and VEGF, as well as blood flow, shear forces and chemokine signaling. Future research will need to identify whether specific endothelial cell types are uniquely endowed with the capacity to form neo-collaterals upon injury, what genetic and epigenetic mechanisms confer the risk to progressively lose collaterals in aging, and how we can devise both preventative and therapeutic measures to maintain and functionalize collaterals to mitigate the most devastating consequences of ischemic disease.

## Author Contributions

TP and HG contributed to conception and design of the mini review. TP performed the literature research and wrote the initial draft of the manuscript. CH and HG provided valuable feedback to the multiple versions of the manuscript and helped edit it. All authors read and approved the submitted version.

## Funding

This work was supported in parts by the Leducq Foundation (Leducq ATTRACT 17 CVD 03), by Deutsche Forschungsgemeinschaft (DFG, German Research Foundation: HA5741/5-1, Project-ID 417284923, and Project-ID 424778381 –TRR 295), and German Federal Ministry of Education and Research (BMBF CSB 01EO1301).

## Conflict of Interest

The authors declare that the research was conducted in the absence of any commercial or financial relationships that could be construed as a potential conflict of interest.

## Publisher's Note

All claims expressed in this article are solely those of the authors and do not necessarily represent those of their affiliated organizations, or those of the publisher, the editors and the reviewers. Any product that may be evaluated in this article, or claim that may be made by its manufacturer, is not guaranteed or endorsed by the publisher.
